# SARS-CoV-2 Infection Risk and COVID-19 Prevalence and Mortality in Cancer Patients During the First Wave of COVID-19 Pandemic in a Virus Epicenter in Northern Italy

**DOI:** 10.3390/cancers17091536

**Published:** 2025-05-01

**Authors:** Matilde Corianò, Luca Isella, Chiara Tommasi, Benedetta Pellegrino, Maria Michiara, Daniela Boggiani, Francesca Pucci, Alessandro Leonetti, Sabrina Bizzoco, Paola Affanni, Licia Veronesi, Elena Rapacchi, Olga Serra, Paolo Sgargi, Giuseppe Maglietta, Antonino Musolino

**Affiliations:** 1 Medical Oncology Unit, University Hospital of Parma, 43126 Parma, Italy; matilde.coriano@unipr.it (M.C.); chiara.tommasi@unipr.it (C.T.); bpellegrino@ao.pr.it (B.P.); michiara@ao.pr.it (M.M.); boggiani@ao.pr.it (D.B.); fpucci@ao.pr.it (F.P.); aleonetti@ao.pr.it (A.L.); erapacchi@ao.pr.it (E.R.); psgargi@ao.pr.it (P.S.); 2 Department of Medicine and Surgery, University of Parma, 43125 Parma, Italy; paola.affanni@unipr.it (P.A.); licia.veronesi@unipr.it (L.V.); 3 SC Oncologia, Ospedale di Circolo Fondazione Macchi, ASST Sette Laghi, 21100 Varese, Italy; luca.isella@asst-settelaghi.it; 4 Local Health Authority of Parma, 43125 Parma, Italy; sbizzoco@ausl.pr.it; 5 Medical Oncology, Breast & GYN Unit, IRCCS Istituto Romagnolo per lo Studio dei Tumori (IRST) “Dino Amadori”, 47014 Meldola, Italy; olga.serra2@studio.unibo.it; 6 Clinical & Epidemiological Research Unit, University Hospital of Parma, 43126 Parma, Italy; gmaglietta@ao.pr.it; 7 Department of Medical and Surgical Sciences, University of Bologna, 40126 Bologna, Italy

**Keywords:** cancer, COVID-19, death, incidence

## Abstract

**Background/objectives**: Cancer patients are more vulnerable to SARS-CoV-2 infection and COVID-19 due to their immunocompromised status. This study aims to evaluate the risk of SARS-CoV-2 infection, as well as COVID-19 prevalence and mortality, in cancer patients during the first wave of the COVID-19 pandemic in a virus epicenter of Northern Italy. **Methods**: This retrospective analysis included 40,148 prevalent cancer patients from the province of Parma, Italy, between February and June 2020. Patients were identified from health system records and classified by cancer subtype, treatment status, and COVID-19 diagnosis. The risk of infection and mortality was analyzed using odds ratios (OR) and hazard ratios (HR). **Results**: Among cancer patients, those on active cancer treatment had a higher cumulative risk of all-cause death (HR 1.83, *p* < 0.000268). Cancer subtype significantly impacted COVID-19 outcomes, with breast cancer patients showing lower incidence and mortality compared to those with lung, colorectal, or bladder cancers. **Conclusions**: Cancer patients, especially those on active treatment, are at increased risk of COVID-19 infection and death. Tailored prevention strategies, including prioritization of vaccination and careful management of cancer treatments, are crucial to mitigate risks during pandemics. These findings provide valuable insights for clinical decision-making in oncological care during public health crises.

## Simple Summary

Cancer patients are more vulnerable to SARS-CoV-2 and COVID-19 due to their immunocompromised status. This study assessed the risk of infection, prevalence, and mortality in cancer patients during the first wave of COVID-19 in Parma, Italy, from February to June 2020. A retrospective analysis of 40,148 cancer patients was conducted, examining cancer subtype, treatment status, and COVID-19 diagnosis. The results showed that cancer patients had a significantly higher risk of infection (OR 1.67, *p* < 0.001). Those on active cancer treatment had a higher risk of all-cause death (HR 1.83, *p* < 0.000268). Cancer subtype influenced COVID-19 outcomes, with breast cancer patients experiencing lower incidence and mortality compared to those with lung, colorectal, or bladder cancers. The findings highlight the need for tailored prevention strategies, prioritizing vaccination and managing cancer treatments during pandemics to mitigate risks for cancer patients.

## 1. Introduction

The coronavirus disease 2019 (COVID-19) pandemic has severely challenged the medical community, causing an unprecedented strain on the community worldwide, especially in the oncology field. Patients affected by cancer are, indeed, purported to be more vulnerable to SARS-CoV-2 infection and COVID-19 [1,2]. Furthermore, COVID-19 is associated with significant severity and mortality in patients with cancer [3,4,5,6].

This is due to the systemic immunosuppressive status caused by malignant tumors and anti-neoplastic treatments in cancer patients which increases the risk of SARS-CoV-2 infection. Conversely, SARS-CoV-2 infection and COVID-19 disease could affect the immune status, cancer disease progression, and routine treatment outcomes of patients affected by cancer [7]. Particularly, it has been shown that the first SARS-CoV-2 infection further deteriorated immunity, stimulated inflammatory response, promoted tumor progression, and reduced the anti–PD-1 treatment effect in cancer patients [8].

As a matter of fact, cancer is a heterogeneous entity, encompassing a wide variety of tumor subtypes, each with unique genetic, molecular, and biological characteristics [9,10]. Hence, the importance of trying and understanding the different possible impacts of COVID-19 diagnosis and SARS-CoV-2 infection on cancer patients depending on the type and stage of their oncological disease.

In this retrospective study, we analyzed the risk of COVID-19 diagnosis in patients with cancer, as well as COVID-19 prevalence and mortality by primary tumor subtype. The study focused on a cohort of patients referred to one of the epicenters of the COVID-19 outbreak in Northern Italy (province of Parma, Emilia-Romagna) from 24 February to 11 June 2020, during the first wave of the COVID-19 pandemic.

## 2. Materials and Methods

The study was conducted within a defined geographic area of interest, specifically the healthcare territory of the Azienda Unità Sanitaria Locale (AUSL) of Parma, Italy, which includes the hospitals of Fidenza, Borgotaro, and the Azienda Ospedaliero-Universitaria of Parma.

We conducted our analysis starting from the population of prevalent cancer patients. These were identified by the presence of the E048 exemption code-indicating individuals officially recognized as having cancer in the Italian National Health System (NHS) electronic records, as well as by their confirmed residency in the province of Parma.

Among our cancer population, we defined patients who sought care at any healthcare service (HCS) within the National Health System in the Province of Parma, Italy, for any medical reason during the study period as “NHS users”. Among cancer patients hospitalized for COVID-19 (i.e., those staying in a hospital for at least 24 h), we classified those undergoing cancer treatment during the study period as “on-cancer treatment”, while “off-cancer treatment” patients were those whose most recent cancer treatment had occurred more than six months before the start of the study period

Patients’ baseline characteristics were summarized in terms of absolute numbers and relative frequencies (percentages) in case of categorical variables, and as mean values ± standard deviation in case of quantitative variables. Clopper–Pearson 95%CIs were used to compare percentages among the “NHS users” and “on-cancer treatment” groups’ variables.

The overall time to COVID-19 and the time to all-causes death were analyzed for their dependence on putative predictors using Cox proportional hazard models. Multivariable models were implemented, and the most parsimonious model was identified using the stepwise backward selection procedure. The cumulative incidence of primary endpoints during follow-up was graphically depicted using Aalen–Johansen curves, and the significance of the differences between the hazard sub-distributions was tested using the Fine–Gray model. For interpretive purposes and to elucidate possible relationships among multiple variables, multiple correspondence analysis (MCA) was performed. The scree plot illustrates the percentage of variance explained by each component. The first two dimensions, orthogonal and independent, were selected to capture the maximum variance. MCA reduces the dimensionality of the dataset and transforms both variables and respondents into factor scores. Calculating factor scores and reducing data dimensions allow for the visual representation of both variables and respondents on a bidimensional plane. To identify which variables best define the two dimensions, a contribution indicator was computed and depicted using a bar plot.

MCA served as an intermediate step in the cluster analysis. Specifically, the factor scores derived from MCA were used in a hierarchical clustering on principal components (HCPC) analysis to classify observations into distinct clusters—that is, groups of patients sharing similar baseline characteristics and exhibiting common patterns in clinical and pathological response variables recorded in this study. This integrated MCA-HCPC approach enhances the interpretability of high-dimensional data by simplifying variable relationships and identifying meaningful patient subgroups, potentially uncovering clinically or prognostically relevant patterns.

All statistical analyses were conducted using R Statistical Software (version 4.2.2).

## 3. Results

**Figure 1:**
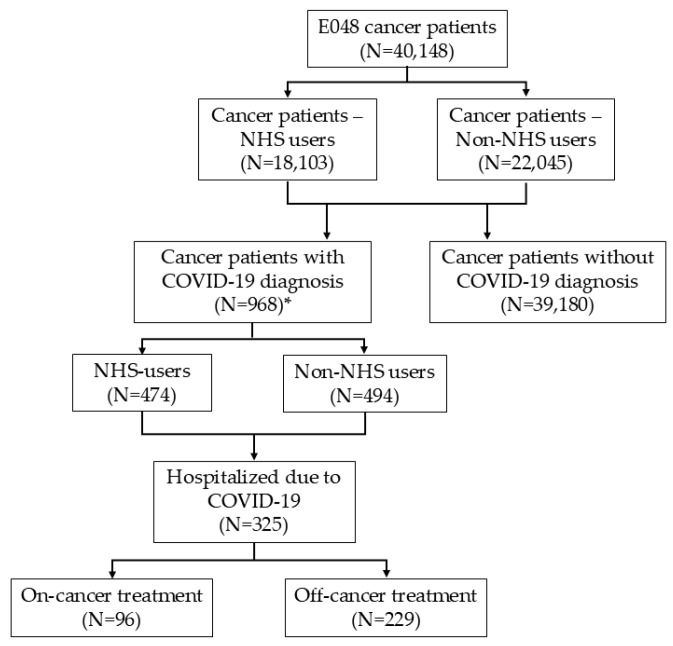
Flow diagram. * Only cancer patients with confirmed residency in the province of Parma and a clinical diagnosis of COVID-19 (irrespective of any SARS-CoV-2-positive tests) were included in the analysis of COVID-19 prevalence and mortality.

The study source population comprised 40,148 prevalent cancer patients, of whom 23,192 (58%) were female and 16,956 (42%) were male. The mean age was 68 years (Figure 1, Table 1).

A total of 968 patients were diagnosed with COVID-19. Among prevalent cancer cases, 18,103 were classified as “NHS users”, of whom 474 had a COVID-19 diagnosis. In total, 325 cancer patients were hospitalized due to more severe forms of COVID-19 disease, including 96 classified as “on-cancer treatment” (Figure 1).

Cancer patients had significantly higher odds of testing positive for SARS-CoV-2 (OR 1.67, 95%CI 1.51–1.77, *p* < 0.001) compared with non-cancer patients. There was a discrepancy between the number of prevalent cancer patients with COVID-19 and those with a positive SARS-CoV-2 test due to the fact that, at that time, according to the Italian Minister of Health’s recommendation [11,12], most COVID-19 diagnoses were based on typical clinical and radiological features, regardless of SARS-CoV-2 test results.

Tumor characteristics of the prevalent cancer cases are summarized in Table 1.

**Figure 2:**
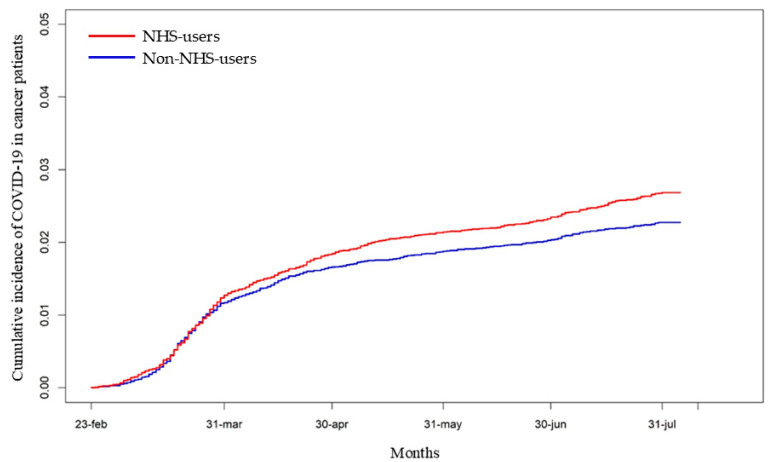
Cumulative incidence of COVID-19 diagnosis among “NHS users” and “non-NHS users” in cancer patients. [HR: 1.18, 95% CI: 1.04–1.34; *p* < 0.011].

The most frequent cancer subtypes were breast (17%), skin (23%, cumulative of melanoma and non-melanoma types), and prostate (8%). “NHS users” had a significantly higher cumulative incidence of COVID-19 compared to “non-NHS users” (HR: 1.18, 95% CI: 1.04–1.34; *p* < 0.011) (Figure 2).

**Figure 3:**
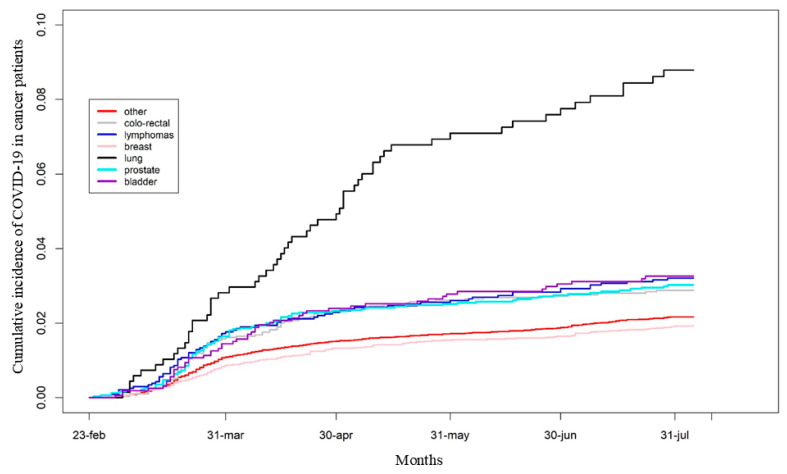
Cumulative incidence of COVID-19 diagnosis in the overall population of cancer patients according to cancer subtypes.

However, the cumulative incidence of COVID-19 by cancer subtype was similar between “NHS users” and the total population of prevalent cancer cases (Supplementary Figure S1 and Figure 3, respectively).

Breast cancer (BC) patients had the lowest cumulative incidence of COVID-19 and a significantly lower HR compared to other cancer subtypes, except for colorectal cancer and other unspecified cancer types (Figure 3 and Table 2).

**Figure 4:**
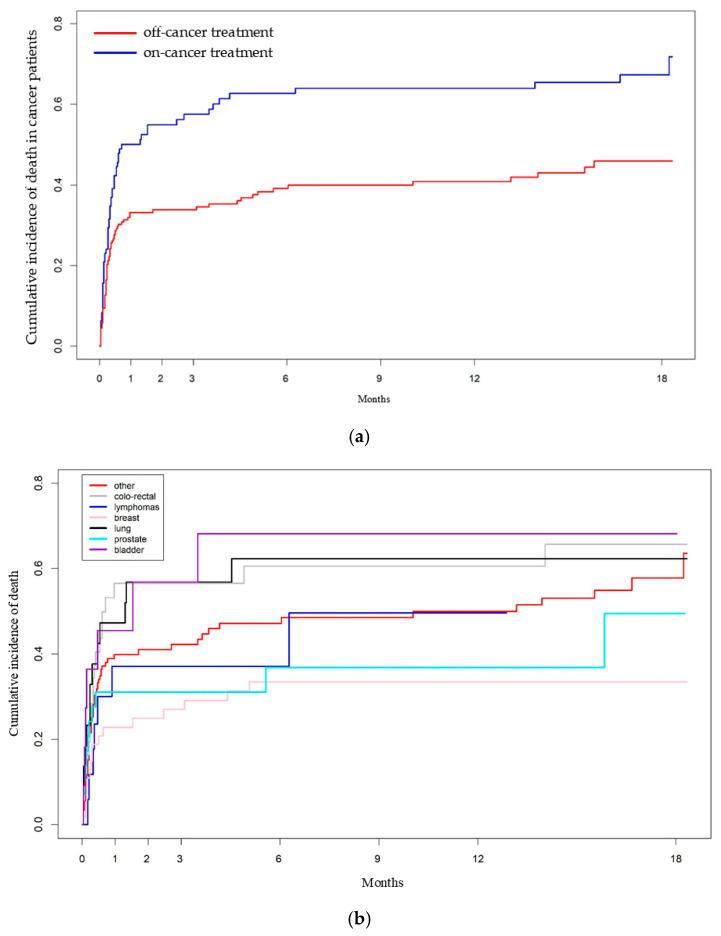
(**a**). Cumulative incidence of all-causes death among the hospitalized cancer patients with COVID-19 diagnosis according to “on cancer treatment” status [HR: 1.83 (95% CI: 1.32–2.53, *p* < 0.000268)]. (**b**). Cumulative incidence of all-causes death among the hospitalized cancer patients with cancer diagnosis with COVID-19 according to cancer subtypes.

Differences in the cumulative risk of all-cause mortality according to “on-cancer treatment” status and by cancer subtype were assessed in the subpopulation of 325 cancer patients hospitalized for COVID-19, and are illustrated in Figure 4a,b.

“On-cancer treatment” patients had a significantly higher cumulative risk of all-cause mortality [HR: 1.83 (95% CI: 1.32–2.53, *p* < 0.000268)] compared to “off-cancer treatment” patients. Additionally, BC patients had a significantly lower HR for mortality compared to all other cancer subtypes, except for prostate cancer and lymphomas (Supplementary Table S1a,b).

We performed a multivariable analysis of the cumulative incidence of all-cause mortality (Table 3).

Bladder and colorectal cancer patients had a higher risk of all-cause mortality compared to BC patients [HR: 4.35 (95% CI: 1.53–12.36, *p* = 0.00587) and HR: 2.60 (95% CI: 1.20–5.27, *p* = 0.0826), respectively]. Patients with later-stage cancer, low/normal lymphocyte and platelet counts, and elevated C-reactive protein (CRP) levels also had a higher risk of all-cause mortality.

**Figure 5:**
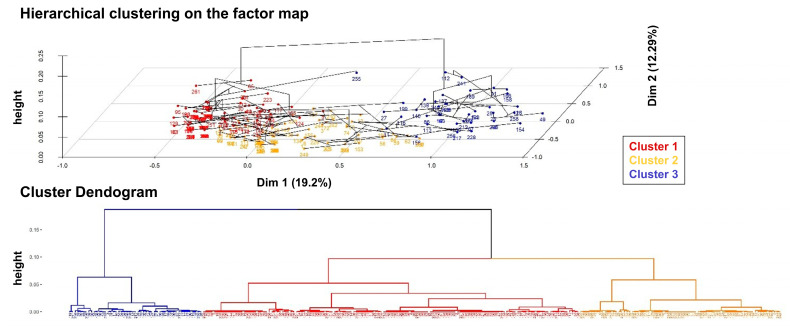
Cluster analysis. On the top, the bidimensional plot from the MCA displays each patient, color-coded according to their assigned cluster, as identified through the HCPC analysis. At the bottom, the dendrogram illustrates the hierarchical clustering process, highlighting the division of the overall sample into three distinct clusters.

MCA was performed on 261 hospitalized patients, considering 15 relevant variables (64 out of the total of 325 patients were excluded due to a high rate of missing data). This analysis revealed that the first two dimensions explain approximately 31.5% of the overall variability (Supplementary Figure S2). The first dimension (19.2%) was primarily influenced by disease stage and “on-cancer treatment” status, while the second dimension (12.3%) was mainly characterized by cancer subtype and age (Supplementary Figure S3). Based on this map, the HCPC analysis identified three distinct clusters (Figure 5).

Supplementary Table S2 provides details on the variables defining each cluster and a description of the patients within each group. Cluster 1 consists of young women (<65 years) with BC, no comorbidities, and COVID-19 recovery. Cluster 2 includes deceased patients with other cancers, predominantly male, at stages I–III, with frequent COVID-19 non-recovery. Cluster 3 comprises “on-cancer treatment” stage IV lung cancer patients with poor radiological responses and high mortality (Figure 5). The results of the cluster analysis are consistent with the multivariable analysis (Table 3).

## 4. Discussion

This study analyzed the incidence, prevalence and mortality of SARS-CoV-2 infection and COVID-19 disease among patients diagnosed with various cancer subtypes in the province of Parma, Emilia Romagna, Italy, one of the epicenter areas of Northern Italy during the first wave of the COVID-19 pandemic.

This work focused on the first wave of COVID-19, at a time when individuals were neither vaccinated nor previously exposed to any SARS-CoV-2 virus. Therefore, these data reflect how the virus affected people with no prior immunity, and are particularly valuable since they could prove useful in the event of future pandemics caused by unknown viruses.

Our findings are the following: (i) the cumulative incidence of COVID-19 was higher among cancer patients who sought care at any healthcare service (“NHS users”) for any medical reason during the observation period; (ii) the cumulative incidence of mortality was higher in patients undergoing active cancer treatment compared to those not receiving active therapies; (iii) different tumor subtypes were associated with varying susceptibilities and outcomes to COVID-19, with BC showing the lowest cumulative incidence and mortality, while lung, colorectal, and bladder cancer exhibited the highest; (iv) we identified three distinct patient clusters based on common characteristics.

These results are consistent with existing literature and highlight the complex interplay between cancer, its treatments, and COVID-19 outcomes.

Previous studies have reported a higher prevalence of COVID-19 among cancer patients compared to the general population. Liang et al. and Kuderer et al. found that cancer patients, particularly those undergoing active treatment, were more likely to contract COVID-19 due to their immunocompromised state and frequent hospital visits for therapy [13,14]. The increased risk observed in our cohort may also be attributed to the overwhelming strain on the healthcare system in Northern Italy at the time, which led to higher exposure, especially for patients receiving immunosuppressive therapies. Additionally, many cancer patients are older and have comorbidities such as cardiovascular disease and diabetes, both of which have been associated with worse COVID-19 outcomes [15]. The higher cumulative incidence of COVID-19 among cancer patients seeking healthcare services in our analysis aligns with the findings of Lee et al., who reported that cancer patients were more frequently exposed to healthcare environments where the virus could spread [1]. Notably, during the early stages of the pandemic, cancer care centers were often overwhelmed, further increasing the risk of nosocomial transmission. Patients undergoing cancer treatments such as chemotherapy required frequent in-person visits to hospitals or outpatient clinics, heightening their exposure to SARS-CoV-2. The necessity of continuing cancer treatments during the pandemic presented a significant challenge for healthcare providers, who had to balance the risk of viral exposure against the potential harm of delaying cancer therapies.

The higher cumulative incidence of death among cancer patients undergoing active treatment, compared to those not receiving active therapy, aligns with findings from Kuderer et al. and Lee et al. Both studies identified chemotherapy and immunotherapy as factors associated with increased mortality in cancer patients with COVID-19 [1,16]. Indeed, active anticancer treatments can significantly compromise the immune system, making patients more vulnerable to viral infections and their complications.

Regarding the observed statistically significant difference in cumulative COVID-19 incidence between ‘NHS users’ and ‘non-NHS users’, this could be explained by two factors: (i) the exclusion of missing data, as some ‘non-NHS users’ may have been absent from the pandemic area during that period; (ii) greater exposure among ‘NHS users’, who sought care at hospitals and medical clinics.

The differential susceptibility to COVID-19 and its outcome based on tumor phenotype is an important observation supported by recent studies [17,18]. Salvatore et al. found that patients affected by colorectal cancer, hematologic malignancies, kidney cancer, and lung cancer had significantly higher rates of hospitalization [18]. These data were consistent with the findings of this study. Conversely, prostate cancer was negatively associated with hospitalization and intensive care unit admission [18].

As regards the mechanisms of the viral infection, it is known that SARS-CoV-2 uses the ACE2 receptor for entry and TMPRSS2 for S protein priming [19]. In this context, ACE2 levels among cancer subtypes may influence COVID-19 susceptibility, and bioinformatic data showed an increased level of mRNA expression of both ACE2 and TMPRSS2 in colorectal and lung cancers, which were found to be more susceptible to COVID-19 [20].

Another factor that could influence COVID-19 susceptibility is the immune system. In fact, the more profound immunosuppression caused by hematologic malignancies and treatments such as stem cell transplants and chemotherapy, and the reduced immune response to vaccines, makes patients with hematologic cancers, particularly those with leukemia or lymphoma, more susceptible to COVID-19 and poorer outcomes compared to those with solid tumors [17,18,19,20,21].

Similarly, the severity of COVID-19 outcomes has been found to differ between solid tumor types according to the immune system status. Patients with lung cancer and those undergoing immune checkpoint inhibitors treatment had particularly high risks of severe COVID-19 outcomes, including hospitalization and death. This may be related to the combined impact of impaired immune function due to both the malignancy and the treatment regimen, as well as to the diagnostic overlap between COVID-19-related and immunotherapy-induced pneumonitis. The shared clinical features, such as cough and hypoxia, can pose a diagnostic challenge for physicians [13,22].

On the other hand, some solid tumor types, such as BC, might have lower risks of severe COVID-19 outcomes, potentially due to being in an earlier stage at diagnosis in most cases, and less aggressive treatments performed with more stable diseases occurring in the metastatic setting [3]. In addition, a possible protective effect of female sex against severe COVID-19 cases cannot be ruled out [23]. In fact, females may possess a potentially more efficient form of the ACE2 receptor, which could reduce susceptibility to SARS-CoV-2 infection, providing women with a relative resistance. A possible role of the gut microbiome composition in women affected by BC as a factor to explain the association of BC with COVID-19 was hypothesized, although this would need further investigation [20].

This study has some strengths and limitations.

This is the only study reporting the prevalence and outcomes of COVID-19 during the first pandemic wave in a robust cohort of patients from Northern Italy’s virus epicenter. It represents a large real-world analysis that enhances our understanding of the impact of COVID-19 on patients with different cancer subtypes, particularly during the first wave of the pandemic, when information was scarce. Additionally, this is a population-based study: we included all prevalent cancer patients within a specific geographic area. To minimize biases due to missing data, we focused on the population that was certainly present in the area during the first wave, using the proxy of at least one HCS access for any reason to distinguish “NHS users” from “non-NHS users”. Nevertheless, the retrospective nature of this study represents a limitation, as it may introduce various biases. Moreover, the limited medical knowledge during the first wave of COVID-19 pandemic may have led to inconsistencies in diagnostic criteria, treatment protocols, and patient management, potentially affecting the generalizability of the findings to later phases of the pandemic. Furthermore, COVID-19 mortality was underreported, and therefore compromised, due to missing events during the first wave; moreover, it was difficult to clarify whether death was due to COVID-19 or to its complications derived from cancer disease progression. This led to the lack of data on COVID-19-related mortality in our analysis. Finally, we did not collect any data regarding non-cancer patients; therefore, it was not possible to make any comparisons between cancer and non-cancer patients, which could be interesting to underline the impact of COVID-19 on people affected by malignant tumors. Likewise, the absence of pre-pandemic data limited the ability to provide meaningful comparative perspectives.

## 5. Conclusions

These data from the first wave of the COVID-19 pandemic may help identify and protect vulnerable cancer patients from exposure to SARS-CoV-2 or future pandemics. It is imperative that cancer care systems continue to implement robust safety protocols, prioritize vaccination efforts, and carefully balance the need for ongoing cancer treatments with the risks of viral exposure. This includes considering the possibility of delaying treatment in low-risk cancer patients receiving adjuvant therapies, particularly during potential future pandemics or resurgences of COVID-19.

## Figures and Tables

**Table 1:** Patients’ baseline characteristics.

Study Population	N = 40,148 (%)
**Sex**	
Female/male	23,192 (58)/16,956 (42)
**Age**	
Mean (SD)	68 years (15.6)
**Any referral to local health services**	
Yes (NHS users)/No (non-NHS users)	18,103 (45)/22,045 (55)
**COVID-19 diagnosis**	
NHS users	474/18,103 (2.5; 95% CI, 2.0–2.4)
Non-NHS users	494/22,045 (2.2; 95% CI, 2.4–2.9)
**Most common cancer subtypes**	
Breast cancer	6844 (17)
Skin cancer (non-melanoma)	5951 (15)
Skin cancer (melanoma)	3235 (8)
Prostate cancer	3155 (8)
Colo-rectal cancer	2843 (7)
Thyroid cancer	1916 (5)
Bladder cancer	1590 (4)
Lymphoma	1427 (4)
Endometrial cancer	1037 (3)
Kidney cancer	980 (2)
Lung cancer	677 (2)
Leukemia	401 (1)
Pancreatic cancer	155 (0.4)
Other subtypes	7425 (18)
Missing/unknown	2512 (6.2)

**Table 2:** Cumulative incidence of COVID-19 diagnosis among the main cancer subtypes compared to breast cancer.

Cancer Subtype	HR	Lower CI	Upper CI	*p*-Value
** Other**	1.166	0.9041	1.504	0.2364
** Colorectal**	1.273	0.851	1.903	0.24038
** Lymphoma**	2.046	1.4488	2.89	<0.001
** Lung**	5.024	3.4261	7.367	<0.001
** Prostate**	1.525	1.0654	2.183	0.02112
** Bladder**	1.929	1.2419	2.996	0.00345

**Table 3:** Multivariable analysis on cumulative incidence of all-causes death. * at COVID-19 diagnosis.

Variables	HR	Lower	Upper	*p*-Value
**“On-cancer treatment” status**	1.45	0.80	2.61	0.22204
**Other cancer subtypes**	1.54	0.89	2.67	0.1272
**Colorectal**	2.60	1.28	5.27	0.00826
**Lymphomas**	2.39	0.74	7.68	0.14414
**Lung**	1.00	0.38	2.63	0.99296
**Prostate**	1.22	0.48	3.10	0.67269
**Bladder**	4.35	1.53	12.36	0.00587
Low neutrophils *	0.40	0.14	1.11	0.07808
Normal neutrophils *	0.88	0.57	1.35	0.56614
Low lymphocytes *	4.36	1.24	15.33	0.02182
Normal lymphocytes *	2.81	0.84	9.36	0.09258
Low platelets *	2.12	1.03	4.38	0.04232
Normal platelets *	1.41	0.79	2.51	0.24595
Low CRP *	0.53	0.08	3.75	0.52668
Normal CRP *	0.00	0.00	0.00	<0.0001
**Clinico-pathological stage**	0.36	0.19	0.68	0.00164

## Data Availability

The data presented in this study are available on request from the corresponding author due to ethical reasons.

## Supplementary Materials

The following supporting information can be downloaded at: https://www.mdpi.com/article/10.3390/cancers17091536/s1. Supplementary Table S1a. Cumulative incidence of all-causes death among hospitalized cancer patients with COVID-19 diagnosis according to “cancer treatment” status; Supplementary Table S1b. Cumulative incidence of all-causes death among hospitalized cancer patients with COVID-19 diagnosis according to cancer subtypes; Supplementary Table S2. Variables describing each cluster. Cla/Mod represents the percentage of patients that have the modality “i” and belong to the cluster “j” and Mod/Cla, the percentage of patients belonging to cluster “j” that show the modality “i”. Supplementary Figure S1. Cumulative incidence of COVID-19 diagnosis among the 18103 “NHS-users” patients according to cancer subtypes; Supplementary Figure S2. Bi-dimensional plot of MCA analysis showed the first dimension along the x-axis and the second dimension along the y-axis. Considering the distance from the barycenter, the proximity to the orthogonal axes and the contribution values (form low values in orange to high values in blue), the variable “Clinical Stage” with its modalities “Stage I–III” and “Stage IV” (from the right to the left) have a relevant contribution to the positive pole of the first dimension, while the variable “Cancer subtype” (from “Other tumor sites” to “Breast”, reading from bottom to top) has a positive contribution to the pole of the second dimension. Variables in gray were analyzed only as supplementary quality variables for descriptive purposes; Supplementary Figure S3. Bar plot of the first two dimensions of the MCA analysis. The dashed red line represents the expected contribution value if uniformly distributed. Variables are ordered from left to right based on their contribution, with higher-contributing variables appearing first.

## Abbreviations

The following abbreviations are used in this manuscript:

COVID-19	Coronavirus disease 2019
AUSL	Azienda Unità Sanitaria Locale
HCS	Healthcare service
MCA	Multiple correspondence analysis
HCPC	Hierarchical clustering on principal components
BC	Breast cancer
CRP	C-reactive protein
