# Supplementary material for: SARS-CoV-2 Infection Risk and COVID-19 Prevalence and Mortality in Cancer Patients During the First Wave of COVID-19 Pandemic in a Virus Epicenter in Northern Italy
Source: Cancers (Basel). 2025 May 1;17(9):1536. doi: 10.3390/cancers17091536 (PMC12070974; doi:10.3390/cancers17091536)
Supplement: Supplementary file 1 [file cancers-17-01536-s001.zip › cancers-3556260-supplementary.pdf]

**Patient status**	**HR**	**lower CI**	**upper CI**	***p*-value**
**"On-cancer treatment"**	1.83	1.322	2.533	0.000268

Supplementary Table S1a. Cumulative incidence of all-causes death among hospitalized cancer patients with COVID-19 diagnosis according to "cancer treatment" status.

**Cancer subtype**	**HR**	**lower**	**upper**	***p*-value**
**Other**	1.91	1.13	3.25	0.01648
**Colo-rectal**	2.55	1.35	4.81	0.00394
**Lymphomas**	1.48	0.65	3.37	0.34757
**Lung**	2.55	1.22	5.32	0.01254
**Prostate**	1.47	0.68	3.21	0.33044
**Bladder**	2.90	1.20	7.01	0.01837

Supplementary Table S1b. Cumulative incidence of all-causes death among hospitalized cancer patients with COVID-19 diagnosis according to cancer subtypes.

**Cluster 1**					
	**Cla/Mod**	**Mod/Cla**	**Global**	***p* value**	**v test**
Alive status	87.16	96.99	56.70	<0.0001	14.48
Recovered from COVID-19	74.71	97.74	66.67	<0.0001	11.65
Negative last cancer radiological response	71.43	93.98	67.05	<0.0001	9.86
Stage I-III cancer	61.40	99.25	82.38	<0.0001	7.90
"Off-cancer treatment" status	64.44	87.22	68.97	<0.0001	6.58
Normal (1000-4800/uL) lymphocytes count	61.68	49.62	41.00	0.00404	2.88
Breast tumor site	67.92	27.07	20.31	0.00404	2.76
Normal (150000-450000/uL) platelets count	55.17	84.21	77.78	0.00404	2.53
Female sex	58.21	58.65	51.34	0.00404	2.39
Age ≤65 years	63.93	29.32	23.37	0.00404	2.30
No comorbidities	73.91	12.78	8.81	0.00404	2.29
Non-smokers	63.79	27.82	22.22	0.00404	2.20
Lung tumor site	27.78	3.76	6.90	0.00404	-2.00
Patients with comorbidities	48.74	87.22	91.19	0.00404	-2.29
Platelets <150000/uL	36.00	13.53	19.16	0.00404	-2.33
Male sex	43.31	41.35	48.66	0.00404	-2.39
Lymphocytes <1000/uL	43.51	50.38	59.00	0.00404	-2.88
Partial or complete response at last radiological follow up	0.00	0.00	4.21	0.00404	-3.60
Ex smoker	31.25	15.04	24.52	0.00404	-3.62
Non-evaluable last cancer radiological response	12.50	3.01	12.26	<0.0001	-4.77
Progressive or stable disease at last radiological follow up	9.30	3.01	16.48	<0.0001	-6.23
"On-cancer treatment" status	20.99	12.78	31.03	<0.0001	-6.58
Stage IV cancer	2.17	0.75	17.62	<0.0001	-7.90

Not recovered from COVID-19	3.45	2.26	33.33	<0.0001	-11.65
Dead status	3.54	3.01	43.30	<0.0001	-14.48
**Cluster 2**					
	**Cla/Mod**	**Mod/Cla**	**Global**	***p* value**	**v test**
Dead status	64.60	85.88	43.30	<0.0001	9.87
Not recovered from COVID-19	71.26	72.94	33.33	<0.0001	9.36
Non-evaluable last cancer radiological response	84.38	31.76	12.26	<0.0001	6.41
Patients with comorbidities	35.71	100.00	91.19	<0.0001	3.98
Other tumor sites	38.95	87.06	72.80	0.00021	3.70
Stage I-III cancer	37.21	94.12	82.38	0.00027	3.64
Male sex	43.31	64.71	48.66	0.00033	3.59
Age >80 years	43.93	55.29	41.00	0.00126	3.23
Ex smokers	45.31	34.12	24.52	0.01457	2.44
Progressive or stable disease at last radiological follow up	18.60	9.41	16.48	0.03013	-2.17
Partial or complete response at last radiological follow up	0.00	0.00	4.21	0.01180	-2.52
Breast tumor site	15.09	9.41	20.31	0.00171	-3.14
Female sex	22.39	35.29	51.34	0.00033	-3.59
Stage IV cancer	10.87	5.88	17.62	0.00027	-3.64
Age ≤65 years	13.11	9.41	23.37	0.00012	-3.85
Non-smokers	12.07	8.24	22.22	<0.0001	-3.95
No comorbidities	0.00	0.00	8.81	<0.0001	-3.98
Recovered from COVID-19	13.22	27.06	66.67	<0.0001	-9.36
Alive status	8.11	14.12	56.70	<0.0001	-9.87
**Cluster 3**					
	**Cla/Mod**	**Mod/Cla**	**Global**	***p* value**	**v test**
Stage IV cancer	86.96	93.02	17.62	<0.0001	12.72
Progressive or stable disease at last radiological follow up	72.09	72.09	16.48	<0.0001	9.36
"On-cancer treatment" status	46.91	88.37	31.03	<0.0001	8.59
Partial or complete response at last radiological follow up	100.00	25.58	4.21	<0.0001	6.16
Dead status	31.86	83.72	43.30	<0.0001	5.89
Lung tumor site	55.56	23.26	6.90	<0.0001	3.89
Neutrophilis <2000/uL	46.67	16.28	5.75	0.00552	2.78
Not recovered from COVID-19	25.29	51.16	33.33	0.00876	2.62
CRP >10 mg/L	17.95	97.67	89.66	0.04678	1.99
CRP ≤10 mg/L	3.70	2.33	10.34	0.04678	-1.99
Normal (150000-450000/uL) platelets count	13.79	65.12	77.78	0.03760	-2.08
Non-evaluable last cancer radiological response	3.13	2.33	12.26	0.01952	-2.34
Unknown procalcitonin levels	2.78	2.33	13.79	0.00944	-2.60
Other tumor sites	12.63	55.81	72.80	0.00901	-2.61
Recovered from COVID-19	12.07	48.84	66.67	0.00876	-2.62
Neutrophils (>2000/uL)	14.63	83.72	94.25	0.00552	-2.78
Unknown smoke habit	8.66	25.58	48.66	0.00089	-3.32
Age ≥80 years	7.48	18.60	41.00	0.00084	-3.34
Alive status	4.73	16.28	56.70	<0.0001	-5.89

"Off-cancer treatment" status	2.78	11.63	68.97	<0.0001	-8.59
Negative last cancer radiological response	0.00	0.00	67.05	<0.0001	-10.43
Stage I-III cancer	1.40	6.98	82.38	<0.0001	-12.72

Supplementary Table S2. Variables describing each cluster. Cla/Mod represents the percentage of patients that have the modality "i" and belong to the cluster "j" and Mod/Cla, the percentage of patients belonging to cluster "j" that show the modality "i".

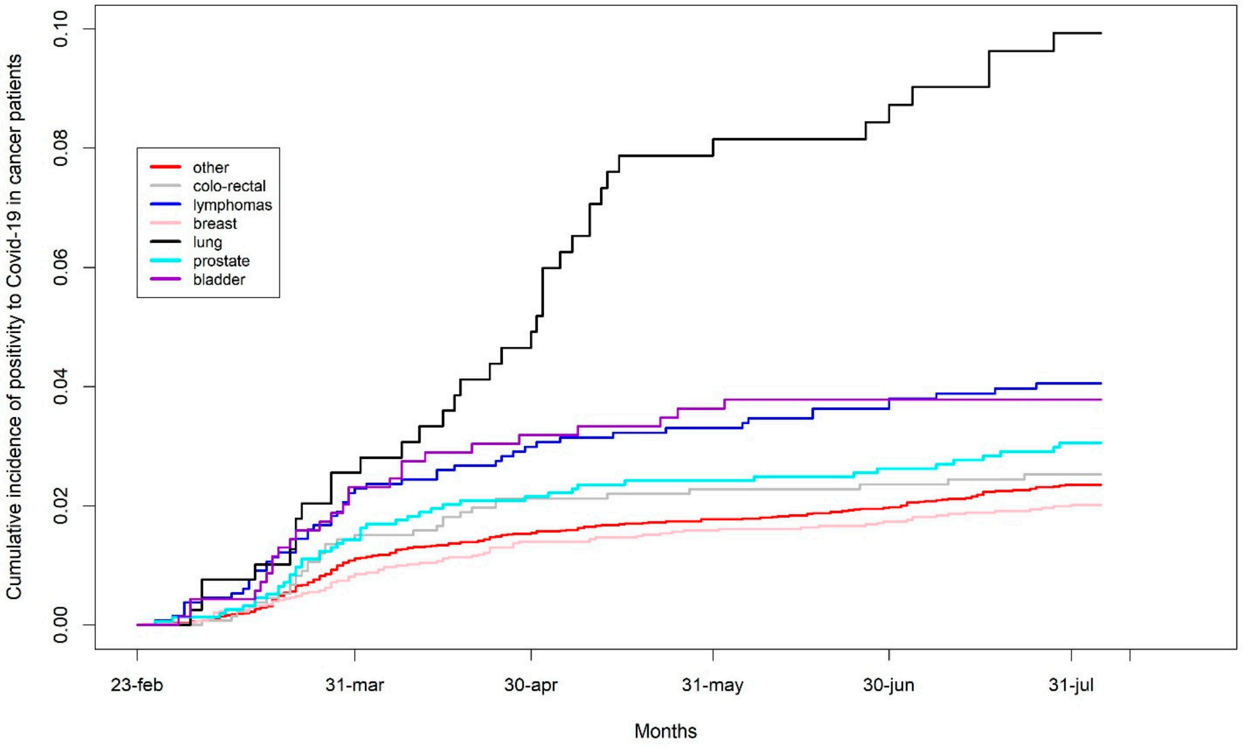

Supplementary figure S1. Cumulative incidence of COVID-19 diagnosis among the 18103 "NHS-users" patients according to cancer subtypes.

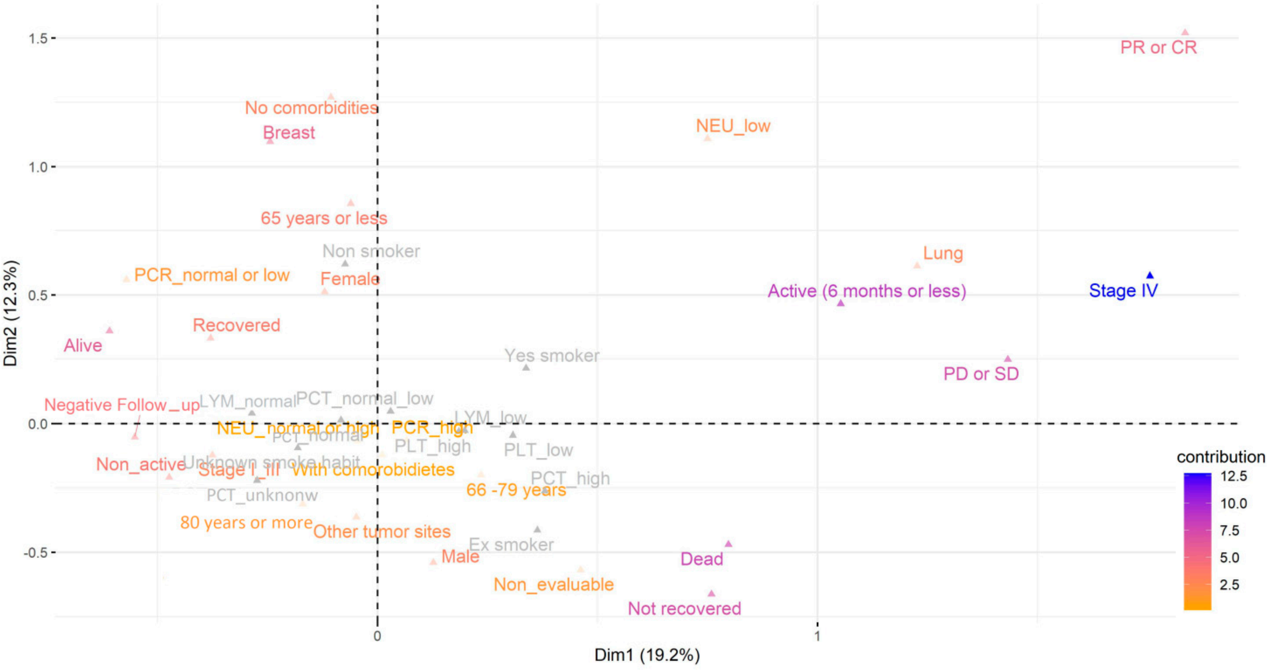

Supplementary figure S2. Bi-dimensional plot of MCA analysis showed the first dimension along the x-axis and the second dimension along the y-axis. Considering the distance from the barycenter, the proximity to the

orthogonal axes and the contribution values (form low values in orange to high values in blue), the variable "Clinical Stage" with its modalities "Stage I-III" and "Stage IV" (from the right to the left) have a relevant contribution to the positive pole of the first dimension, while the variable "Cancer subtype" (from "Other tumor sites" to "Breast", reading from bottom to top) has a positive contribution to the pole of the second dimension. Variables in gray were analyzed only as supplementary quality variables for descriptive purposes.

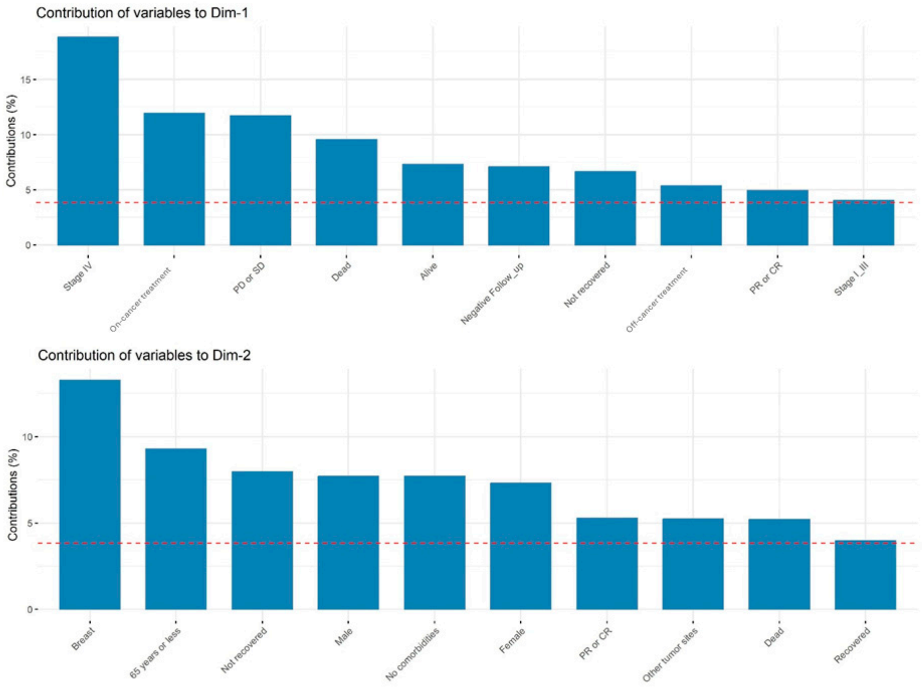

Supplementary Figure S3.

Bar plot of the first two dimensions of the MCA analysis. The dashed red line represents the expected contribution value if uniformly distributed. Variables are ordered from left to right based on their contribution, with higher-contributing variables appearing first.
